# Lack of patients? – a hypothesis for understanding discrepancies between hospital resources and productivity

**DOI:** 10.1186/1472-6963-6-42

**Published:** 2006-04-02

**Authors:** Dag Bratlid

**Affiliations:** 1 Institute of Laboratory medicine, children's and women's diseases, The Norwegian University of Science and Technology, Department of paediatric and adolescent medicine, St. Olavs University Hospital, Trondheim, Norway

## Abstract

**Background:**

Despite a substantial increase in hospital resources, increased hospital admissions and out-patient visits, long waiting lists have been a significant problem in Norwegian health care. A detailed analysis of the development in resource allocation and productivity at St. Olavs University Hospital in central Norway was therefore undertaken.

**Methods:**

Resource allocation and patient volume was analysed during the period 1995 to 2001. Data were analysed both for emergency and elective admissions as well as outpatient visits specified into new referrals and follow-up consultations.

**Results:**

Full time employee equivalents for doctors and nurses increased by 36.6% and 25.9%, respectively, and all employees by 28.1%. However, admitted patients, outpatient consultations and surgical procedures only increased by 10%, 15% and 8.3%, respectively. Thus, the productivity for each hospital employee, defined as operations pr. surgeon, outpatient consultations pr. doctor etc. was significantly reduced. A striking finding was that although the number of outpatient consultations increased, the number of new referrals actually went down and the whole increase in activity at the outpatient clinics could be explained by a substantial increase in follow-up consultations. This trend was more evident in the surgical departments, where some departments actually showed a reduction in total outpatient consultations.

**Conclusion:**

In view of the slow increase in hospital activity in spite of a significant increase in resources, it can be speculated that patient volume might be a limiting factor for hospital activity. The health market (patient population) might not be big enough in relation to the investments in increased production capacity (equipment and manpower).

## Background

The Norwegian National Health Service has the last years been through a period of substantial growth. The Health Service's share of the gross national product increased from 8.2 to 9.5 percent during the period 1995–2004 [1]. When health care costs are calculated in USD and corrected for purchasing power, only United States and Switzerland have higher health care costs than Norway [1]. However, while government spending on health care is only 44% in the U.S., and 54% in Switzerland, 84% of health care in Norway is covered through tax revenues.

Norway has a rather healthy population of 4.6 million people. Perinatal mortality is low, average life span is high, and socio-economic figures (unemployment rate, average income) are among the best in the world [1]. All in-hospital care is provided free of charge, and in principle, all other necessary health care is also free, with a maximum accumulated fee for out-of hospital services (including expensive prescription drugs) of approximately USD 250 per person and year.

Simultaneously, the gap between hospital resources and patients demand for treatment does not seem to be reduced. From the patients' view, the health care is characterised by never ending waiting lists particularly for surgical procedures, and sometimes completely lack of treatment for certain conditions. As seen from the hospitals, in spite of the substantial allocation of government money to hospital financing, the long and often increasing waiting lists might still be explained by lack of manpower and economic resources [2].

The performance of the Norwegian National Health Service has traditionally been analysed by national (macro) data [3] and gross data from hospitals, to a lesser extent by analyses of data from individual departments [4,5]. Macro-analyses will, however, often conceal variations in developmental trends between individual departments and hospitals. Such variations might also have opposite directions than the over all picture. In-depth analyses on the micro-level can therefore not only give additional insight into the nature of the problems encountered, but also lead to a deeper understanding of possible mechanisms. Furthermore, macro-analyses on a national level are often considered not to be precise enough by administrators and directors at institutional and departmental levels, who often consider their own institution and department to be so unique that national data will not be relevant as a basis for changes. This often results in poor compliance to measures taken by the government on such basis to improve the productivity of hospitals. The understanding of problems related to hospital productivity is therefore often based on pseudo-evidence data.

On this background a detailed analysis of the development in resource allocation and productivity at St. Olavs University Hospital in central Norway has been undertaken, with a special focus on case-mix. St. Olavs University Hospital is a 950-bed university affiliated teaching hospital and the 3^rd ^largest hospital in the country with services in all major medical specialities except organ transplantation and paediatric cardiac surgery. The catchment population is represented by the 266323 people (2001, up 3.2% from 1995) in 25 communities in the county of Sør-Trøndelag. The hospital is also the regional (referral) hospital for the counties of Nord-Trøndelag (population 127 457) and Møre og Romsdal (population 243 855) for services offered at university hospitals only, such as cardiac surgery, neurosurgery, level 3 neonatal intensive care, paediatric oncology, and a few others. The hospital also is the location for three of altogether 41 nationally centralised low volume, highly specialised services, namely advanced prenatal invasive treatment, neonatal surgery and photophoresis, These services account for less than 100 admissions per year.

### Purpose of the study

The purpose of the study was to analyse patient volume figures in relation to available resources as a measure of productivity, in view of possible changes in case mix and simple quality criteria in a large general hospital. Since most of the activity in a hospital (both patient care and resource expenditure) is generated on the micro-level where the patient meets the doctor, complete analysis of different departments and health care professions were considered important.

## Methods

### Study period

Norwegian hospitals have traditionally been owned and operated by 19 different county councils. Most of the funding was provided by the national government in the form of block grants based on the size of the population as well as demographic and social characteristics. Since1997, the block funding has been gradually replaced by a matching grant depending on the number and composition of treatments (DRG-based), from 30% of gross budget in 1997 to 60% in 2001. Outpatient activities are reimbursed based on activity (number of consultations). Furthermore, because of the problems within the Norwegian National Health Service, a reform was passed where the central government took over the operation of all hospitals organised through five regional hospital enterprises, active from January 2002. The analysis of St. Olavs University Hospital was therefore focused on the years from 1995 to 2001 leading up to this reform.

### Data acquisition and analysis

Data on departmental health care personnel, economic resources, patient admissions and outpatient consultations were obtained from the yearly hospital statistical reports as well as the monthly reports submitted by the hospital to the National Patient Registry. Data on admissions and in-hospital-mortality of high risk patients groups (DRG 14 (stroke), DRG 89–90 (Pneumonia in patients > 17 years) DRG 121–122 (Myocardial infarction), DRG 127 (heart failure and shock), DRG 210–211 (Hip fracture in patients > 17 years) and DRG 236 (Hip and pelvic fracture)) were obtained from the National Patient Registry and analysed and compared by a year-to-year comparison as well as for the whole study period.

Data were analysed both for emergency and elective admissions as well as outpatient visits specified into new referrals and follow-up consultations. A new referral was defined as a patient having his first consultation after referral for a specific (new) problem. A follow-up consultation was defined as a consultation for a problem for which the patient had been seen before during the last 12 months. These two patient groups are accounted separately and therefore specified in all reports. It was postulated that such analyses of a case report from departments at a single institution could identify developmental trends not observed in the national data, and thereby generate new hypotheses for the explanation of the seemingly discrepancy between resource allocation and productivity.

## Results

### Total hospital resource expenditure

Table 1 shows key numbers for the increase in hospital employees for the years 1995 to 2001, detailed for the different medical professions. There was a substantial increase in the number of full time equivalent employees (FTEs) during these years, which in total increased by 28.1% as calculated from the last day of the year. Contract labour has never been of any significance and was not included in the analysis. There was particularly an increase in office and administrative personnel (49.9%), doctors (36.6%) and nurses and nurse assistants (29.9%). The increase in the number of radiology technicians (23.6%) technical personnel (22.2%) and medical laboratory technologists (21.7%) was somewhat lower. As seen from Table 1, the total costs of running the hospital increased substantially over the same time period, particularly with regard to salary expenses (90.1%). From Table 1 it can be calculated that the average salary expense pr. FTE increased by 48.3%, compared to a national average salary increase of 34.8% during the same time period [6]. There was also a substantial increase in general running costs of 56.1%, compared to an increase in the national consumer price index of 18.3% during the same time period [7].

### Patient volume

As also shown in Table 1, during the same time period there has also been an increase in admitted patients and consultations at the outpatient clinics, but to a considerably lesser extent. Hospital admissions increased by only 10.0% mainly related to an increase in elective admissions. Emergency admissions increased by only 6.4% and emergency admissions as a percentage of total admissions were reduced by 4.8%. In comparison, the catchment population increased by 3.2% during the study period. Furthermore, the average length of stay decreased by 16.4% resulting in a decrease in in-hospital days by 2.4%. Outpatient consultations (including day patients) increased by 15%. However, this was entirely caused by a 31.8% increase in follow-up consultations, while new referrals were actually reduced by 6.6%. During the time period 1995–2001 also he number of operations increased by 8.3% and the number of intensive care patients (excluding neonatology) increased by 15.4%.

### Relative patient load for different professions and activities

In Table 2 these developmental trends are specified by profession and treatment activities. As expected, the greater increase in hospital staffing compared to the increase in patient load resulted in significant reductions in productivity pr. hospital FTE. The number of outpatient consultations pr. doctor pr. year was reduced by 15.7%, the number of surgical procedures pr. doctor pr. year by 20%, and the number of anaesthetised patients and hours with anaesthesia pr. anaesthesiologist pr. year by 19.2% and 13.2% respectively. Also in the medical service professions (laboratory medicine and radiology) the relative workload pr. FTE seemed to be reduced. These developmental trends are further analysed in Tables 3, 4, 5.

### Interdepartmental variations in FTEs

As can be seen from Table 3, the increase FTEs was not evenly distributed between the different departments. The increase in doctors and nurses was mainly seen in the medical departments, with an increase in doctors from 20% to 86.1% (average increase 62.2%) and nurses from 8.8% to 67.2% (average increase of 45.4%). In comparison, the increase in FTEs in the surgical departments, were on average 23.2% for doctors and 14.2% for nurses. During the study period the weekly basic working hours were 38 hours for physicians in call systems and 40 hours for physicians with day work only. Average scheduled extended working hours for physicians were approximately 8 hours pr. week with no major changes during the study period. Nurses working shifts had a 35.5 hour week, others 38 hours per week.

### Interdepartmental variations in patient load and activities

As shown in Table 4 and Table 5 there were also significant differences in the development of patient related activities during the study period, both between different departments but particularly between medical and surgical departments. In most of the medical departments there was an increase in admitted patients, particularly in elective admissions, as well as in total outpatient consultations, both in new referrals and follow-up consultations. The exception is in paediatrics. Some departments also showed significant year to year variations. The increase in new referrals in oncology from 1999–2001 was probably related to a significant increase in radiation capacity. The increase in admissions and reduction in outpatient consultations at the department of dermatology starting in 1997, was related both to a shift in treatment policy and increased capacity. The increase in elective neurologic admissions from 1999 to 2001 was a result of an effort to reduce waiting time that were among the longest in the hospital (average waiting time for elective admission > 1 year).

In most of the surgical departments, however, the increase in admitted patients was more modest, particularly in emergency admissions, and some departments had a significant reduction in emergency admission (ophthalmology, gynaecology and neurosurgery). Also in general surgery and orthopaedic surgery the increase in admitted patients were below the hospital average. In otorhinolaryngology and gynaecology there was no increase or even a reduction in total admissions.

Outpatient consultations in the surgical departments also showed a different pattern than in the medical departments. The increase in the total number of consultations was more modest, and there was a significant decrease in the number of new referrals in many of the departments, in general surgery by 21.2%, in orthopaedic surgery by 14.8%, in gynaecology by 27.1% and in ophthalmology by 33.2%. In several of these departments (and in paediatrics, Table 4), the increase in the total outpatient consultations can completely be explained by a compensatory increase in follow-up consultations, which in some departments was very high, such as general surgery 71.7%, gynaecology 61.3% and paediatrics 52.0%. Furthermore, even in departments with an increase also in new referrals, the number of follow-up consultations increased even more.

### Patient complexity and quality considerations

In Table 6 the number of admitted patients and outcome of the five most important conditions resulting in in-hospital deaths in Norway [8] is given. This includes heart failure/shock (DRG 127), myocardial infarction (DRG 121–122), pneumonia/pleuritis > age 17 (DRG 89–90), stroke (DRG 14) and hip and pelvic fracture (DRG 210–11, 236). Neither the number of patients admitted in these high risk groups nor the outcome (in-hospital deaths) changed during the study period. As also shown in Table 6, the average volume of diagnostic patient work-up for in-hospital patients, such as the number of clinical chemistry laboratory tests, microbiology test and imaging procedures, did not change or was slightly reduced. Thus, none of these data indicate any significant changes in service output during the study period.

## Discussion

The present study shows that the increase in hospital resources far exceeds the increase in patient volume. A higher number of employees and particularly more doctors seem to be needed to treat each patient. Thus, if effectiveness and productivity for medical doctors in 2001 had been at the same level as in 1995, it might be speculated that they could have taken care of a hospital with on average 1110 in-hospital patients with the same case-mix, instead of only 793. One should, however, be careful in generalising results from one hospital, since national as well as international studies have shown that the consumption of hospital resources varies between different geographical regions [3,9]. However, also in the present study some unexplained variations occur. Thus, there is no obvious explanation for the reduction in new referrals from 1999–2001, after a steady increase (Table 1). In 2002 new referrals had again increased to 100713 of a total volume of 281904 out-patient consultations (data not shown), still a reduction of 2.8% from 1995. The trend of an increased follow-up as a main driving force for total out-patient activity therefore seems to hold, particularly since this also was a steady trend in several departments (Table 4, Table 5). Furthermore, the macro-data from the present study is in agreement with national data for the same time period, with the greatest increase in activities seen in the medical departments [3], while surgical activities have been surprisingly stable, and even reduced for some departments.

Ashby and Altman studied hospital productivity during the period 1980–1989 by means of aggregate productivity, defined as the ratio of admissions (after adjusting for the complexity of the patients and outpatient activities) to FTEs [10]. They found that while admissions and FTEs increased with an average of 1.4% and 1.7% per year respectively, aggregate productivity fell with an average of 0.4%. However, by also taken into account intensity of services and changes in intermediate productivity (defined as ratio of services to FTEs) they concluded that hospitals had become more efficient during the study period. However, no analysis of individual hospitals nor raw productivity data were given. Furthermore, even if the yearly increase in admissions in their study was similar to that in the present (1.4% vs. 1.6%), the increase in FTEs were much lower than in the present study (1.7% vs. 4.7%). This difference also supports the speculation that the (aggregate) productivity of St. Olavs Hospital was significantly reduced during the study period.

In a study of trends in structure, productivity, effectiveness and unit costs of hospital and community health services in England in 1997–1999 [12] it was concluded that productivity had grown by a compound rate of 1.9% annually. The general trend did, however, conceal wide fluctuations. Furthermore, the trends were quite different from those found at St. Olavs Hospital, with a reduction in support staff (at St. Olavs Hospital these groups showed a considerable growth), and a reduction in unit labour costs, which at St. Olavs Hospital had risen significantly during the study period.

Based on the present findings several hypotheses might be generated and discussed to explain these developmental trends.

### Increased focus on quality

Hospital performance and expenditure is not only related to patient volume but also to quality and intensity of treatment, as well as case-mix [10,11]. It might therefore be argued that the increase in resource allocation could be related to improvement in treatment quality or changes in case-mix. Thus, during the study period there was an increased focus on quality, particularly in documentation of patient records and reports. This can partly explain the increase in office and administrative staff. On the other hand, admissions of high risk patient groups did not change, neither did in-hospital mortality for these patients, indicating that the severity of sickness or volume of these patients had not changed during the study period [11]. Furthermore, the volume of diagnostic patient work-up, such as laboratory tests and imaging procedures were not increased during the study period, also indicating that complexity and case-mix of admitted patients had not been significantly changed. Thus, data related to treatment quality did not indicate significant changes in case-mix or treatment quality during the study period.

### Bottlenecks and technological developments

Low productivity in hospitals has often been explained by bottlenecks, particularly in the medical service professions such as radiology and anaesthesiology [13,14]. Since a reduction in these activities was seen both in volume/physician (laboratory services, radiology and anaesthesiology) and volume/patient (laboratory services, radiology) it is unlikely that these services were true bottlenecks in the treatment lines.

Also, during the study period no major technological developments or new diseases were introduced, although an increase in the use of cardiovascular stents and laparoscopic surgery was seen. The average patient treated in 2001 was therefore probably not more complicated to treat than in 1995.

### Imbalance between hospital beds and medical resources

Another explanation that might be considered is related to the steady demand by hospital owners for increased effectiveness, reflected in a significant reduction in in-hospital days and average length of stay for each patient. It might be speculated that this trend actually creates more work with each patient by creating a need to see the patient again (in the department or at the outpatient clinic), because the work-up or treatment could not be fully completed during the short hospital stay. This hypothesis is also supported by national data showing that during the period from 1996 to 2003 the number of patients readmitted as an emergency case within 30 days after discharge increased from 8% to 9,6% of all emergency admissions [15]. Furthermore, the number of patient with a single admission during a year was reduced from 57 to 55 per cent of the total number of admission [15]. In a recent European study it was found that 24% of patients admitted to a department of internal medicine were readmitted within 6 months from discharge, with major impact on resource utilisation [16]. One might therefore speculate if the reduction in the number of hospital beds as a measure to increase effectiveness (by reducing the length of stay), has actually resulted in an imbalance between staffing resources and available beds. It can therefore be hypothesised that if the hospital to some extent also had increased the number of beds in relation to the increase in manpower, instead of reducing them by 2% (Table 1), the productivity per employee might also have been increased in stead of being reduced.

### Lack of professional continuity in patient care

There might also be organisational reasons for this development. With the increase in the number of doctors and interrupted working plans, it is difficult to organise the service so the physician who will be seeing the patient at the follow-up consultation at the outpatient clinic is the same who treated the patient while in the department. In the eye of the new doctor, the patient will also be new. This situation is likely to result in the schedule of another follow-up visit before the patient is referred back to the general practitioner. The (new) doctor might feel that this is necessary, but by medical criteria it might not be indicated. Private practitioners in Norway have thus complained that the hospitals keep referred patients too long with too many follow-up visits [4]. This emphasises the need to have clinical guidelines (departmental or national) structuring necessary follow-up, particularly for major patient categories. This is an issue of such impact on hospital productivity and effectiveness that it should not be up to the individual doctor (often in-training) to decide.

### Do hospitals lack patients?

The most striking finding in this study is the data concerning the outpatient population. The significant increase in the number of follow-up consultations, while new referral consultations actually went down must also raise the hypothesis that the slow increase in patient-related activities at the hospital, in spite of a significant increase in resource allocation, can be explained by a relative lack of patients. It might also be speculated if the relatively slow increase in the number of patients who are admitted might be explained in the same way. Data from the Norwegian Patient Registry show that from 1999 to 2000 the number of individuals admitted to Norwegian hospitals increased by 0,3%, while the number of admissions increased by 1,8% [4]. The increased number of admissions is therefore to a large extent caused by re-admittance of patients recently discharged, and not by new patients taken in. It can be speculated that this reflects that the market (patient volume) might not be big enough in relation to the investments in increased production capacity (equipment and manpower) at hospitals. In this context the patient population might actually be a limited reserve. In agreement with this speculation, more hospitals and departments in Norway now advertise their services to patients in other health regions, possibly in order to recruit patients to keep up their activity. This is particularly evident in relation to surgery.

Hospital owners and politicians have for many years asked hospitals to increase their admissions and out-patient consultations to meet the seemingly unlimited demand for treatment. It might therefore be speculated that if patient population (volume) might be limited, the only possible answer to increase volume is to recycle the patients you already have. The modest increase in admitted patients and operations at the surgical departments, but significant increases in follow-up patient at the surgical outpatient clinics, might therefore reflect that it is easier to recycle a successfully operated patient for an extra consultation at the outpatient clinic than to readmit him. Most medical departments also show relatively similar developments in admitted patients and outpatients consultations. It can thus be speculated if the long waiting lists at St. Olavs University Hospital as well as at other Norwegian hospitals, are mainly caused by a tendency to readmit recently treated patients and once again see follow-up patients, instead of scheduling new patients from the waiting lists [17].

Finally, the fact that the patient population over time might be limited can also be related to general social-demographic trends. Although the general picture seems to be an increased demand for new treatments for new diseases or malfunctions, the other trend is an increasingly healthier population, partly because of increased focus on physical exercise, healthier food habits and less smoking. Focus on external hazards, such as traffic accidents have in spite of a significant increase in the number of automobiles resulted in a reduction in fatal accidents by 10% during the last ten years, while injuries have not increased more than the population size [18]. Furthermore, the technological developments, which have made a major impact on hospital treatment, have also made it possible for patients to take care of and monitor their treatment in their own home and reduce the need for hospital visits [4]. This developmental trend will probably continue.

## Conclusion

Based on the gap between the increase in hospital resources and treated patients at St. Olavs University Hospital, it can be hypothesised that the patient population might be a limited reserve. For some departments and specialities the patient volume might not be sufficient to justify the increase in resource allocation. A demand for more resources because of long waiting lists and low productivity should be carefully analysed before the demand is met. In-hospital and inter-hospital redistribution of resources might in some cases be more relevant than an increase in total resources. Waiting list may be more related to system malfunction than patient overload [19,20].

## Competing interests

The author(s) declare that they have no competing interests.

## Authors' contributions

DB is the sole author of this manuscript

## Pre-publication history

The pre-publication history for this paper can be accessed here:



## Figures and Tables

**Table 1:** Full time employee equivalents (FTEs), patient volume and economic data from St. Olavs University Hospital during the time period 1995–2001.

	**1995**	**1997**	**1999**	**2001**	**Change %**
***Personnel***					
Medical doctors	373,25	409,25	487,8	509,8	+36.6
Ratio consultants:interns/residents	1.9	2.0	1.8	1.7	-10.,5
Nurses*	1596.42	1625.6	1805.47	2009.2	+25.9
Ratio nurses:doctors	4.3	4.0	3.7	3.9	-9.7
Midwifes	53	57	66.5	68	+28.3
Radiology technicians	63.25	63.25	66.25	78.2	+23.6
Medical laboratory technologists	205	209.5	213	249.5	+21.7
Office and commercial staff	358.32	383.97	438.1	536.95	+49.9
Technical staff	124.8	142.8	154	155.2	+24.4
Total FTEs	3337.41	3445.31	3739.07	4276.4	+28.1
***Patients***					
Hospital beds	958	957	958	939	-2.0
Admitted patients	40 330	41 769	42 995	44349	+10.0
Emergency	25 005	25 897	26 657	26 606	+6.4
Emergency (%)	62	62	62	60	-4.8
Elective	15 325	15 872	16 338	17 793	+16.1
In-hospital days	296707	292883	291023	289586	-2.4
Average length of stay (days)	7.8	7.5	7.2	6.6	-16.4
Waiting list for admittance	3313**	3 414	3 497	3 118	
Out-patients consultations	234 890	236 423	250 617	270 142	+15.0
New referrals	103 661	108 342	114 079	97 156	-6.6
Control consultations	131 229	128 081	136 520	172 986	+31.8
Waiting lists outpatient clinics	18828**	21 779	18 697	22 355	
Surgical procedures^§^	20 488	22 012	21 199	22 179	+8,3
High level intensive care patients	480	554	567	554	+15.4
Imaging examinations	154 074	154 606	158 477	162 452	+5.4
Laboratory tests and procedures#	3 276 202	3 407 737	3 364 637	3 806 295	+16.2
***Economical data***					
Total operational costs^Φ^	1 575 582	1 928 383	2 260 494	2 803 473	+77.9
Salary costs	1 012 869	1 289 988	1 530 385	1 925 255	+90.1
Running expenses	562 713	638 395	730 109	878 218	+56.1

*includes registered nurses and nurse assistants.

**data from 1996

^§^excluding c-sections and emergency room operations

#includes all tests/procedures at the departments of medical biochemistry, microbiology, immunology and transfusion medicine, pharmacology, and all biopsies and cytological analysis at department of pathology.

^Φ^in Norwegian kroner, thousand

**Table 2:** Relative patient load for different personnel groups at St. Olavs University Hospital in1995 and 2001. No corrections have been made for holidays, other leave or vacancies.

	**1995**	**2001**	**Change %**
Admitted patients/doctor/year	101	87	-13.9
Admitted patients/nurse/year	24	22	-8.3
Admitted patients/office staff/year	106	83	-21.7
In-hospital patients at all hours	813	793	-2.5
In-hospital patients at all hours/doctor	2,2	1,6	-28.3
In-hospital patients at all hours/nurse	0.5	0,39	-20.0
In-hospital patients at all hours/office staff	2,3	1.5	-34.8
Deliveries/midwife/year	57	44	-22.8
Out-patients consultations/doctor/year	629	530	-15.7
Out-patients consultations/nurse/year	147	134	-8.8
Out-patients consultations/office staff/year	656	503	-23.3
Surgical procedures/doctor/year – total*	160	128	-20.0
General surgery	151	134	-11.4
Orthopedic surgery	189	155	-18.0
Neurosurgery	98	100	+2.0
Otorhinolaryngology	177	125	-29.4
Ophthalmology	135	152	+12.6
Gynecology	109	71	-34.9
Anesthetized patients/anesthesiologist/year^§^	661	534	-19.2
Hours with anesthesia/anesthesiologist/year	1 322	1 148	-13.2
Imaging procedures/radiologist/year	7 003	5 198	-25.8
Imaging procedures/radiology technician/year	2 436	2 077	-14.7
Lab test and procedures/technologist/year#	15 981	15 256	-4.5

*number of surgeons calculated as the sum of surgeons at department of general surgery, department of orthopedic surgery, department of neurosurgery, department of otorhinolaryngolgy, department of ophthalmology, and department of obstetrics and gynecology.

^§^calculated on the basis of total number of anesthetized patients

# includes all tests/procedures performed at the department of medical biochemistry, department of microbiology, department of immunology and transfusion medicine, department of pharmacology, and all biopsies and cytological analysis at department of pathology.

**Table 3:** Increase (FTEs) in medical (doctors) and nursing staff (registered nurses and nurse assistants) at selected departments at St. Olavs University Hospital during the time period 1995–2001.

		**1995**	**2001**	**Change %**
***Medical departments***				
Internal medicine	Doctors	48.25	89.8	+86.1
	Nurses	288.25	482.0	+67.2
Oncology	Doctors	14	20,6	+47.1
	Nurses	79	108.85	+37.8
Pediatrics	Doctors	22	32,6	+48.2
	Nurses	124.75	147.25	+18.0
Rheumatology	Doctors	6	7,2	+20.0
	Nurses	22.5	24.5	+8.8
Dermatology	Doctors	6	9	+50
	Nurses	23	27	+17.4
Neurology	Doctors	12	16.4	+36.7
	Nurses	26	30	+15.4
***Surgical departments***				
General surgery	Doctors	40	48.85	+22.1
	Nurses	204.15	221.85	+8.7
Orthopedic surgery	Doctors	30	31.20	+4.0
	Nurses	167.5	188.0	12.2
Neurosurgery	Doctors	10	12	+20
	Nurses	53.5	70.15	+31.1
Gynecology & Obstetrics	Doctors	24.5	33.6	+37.1
	Nurses	160.25	177.90	+11.1
Otorhinolaryngoly	Doctors	10	14	+40
	Nurses	31.75	37.5	+18.1
Ophthalmology	Doctors	13	14.8	+13.8
	Nurses	33.75	35.7	+5.8
Anesthesiology	Doctors	29	38,4	+32.4
	Nurses	138	170.06	+23.2

**Table 4:** Patients admitted, outpatient consultations and case-mix at selected medical departments at St. Olavs University Hospital during the time period 1995 to 2001.

**Department**	**1995**	**1997**	**1999**	**2001**	**Change (%)**
***Internal medicine***					
Total admittance	9 752	10 230	10 924	11 146	+14.3
Emergency	8 972	9 616	9 941	10 143	+13.1
Elective	780	614	983	1 003	+28.6
Outpatient visits, total	26 886	28 425	35 707	41 368	+53.9
New referrals	10 207	11 901	15 394	15 235	+49.3
Control consultatiens	16 679	16 524	20 313	26 133	+56.7
***Oncology***					
Total admittance	1 860	2 008	2 332	2 347	+26.2
Emergency	353	402	513	563	+59.5
Elective	1 507	1 606	1 819	1 784	+18.4
Outpatient visits, total	5 928	6 010	7 824	9 343	+57.6
New referrals	525	637	515	983	+87.2
Control consultatiens	5 403	5 373	7 309	8 360	+54.7
***Pediatrics***					
Total admittance	2 910	3 104	3 129	3 271	+12.4
Emergency	1 950	1 924	2 003	2 098	+7.6
Elective	960	1 180	1 126	1 181	+23.0
Outpatient visits, total	11 208	11 669	13 477	14 539	+29.7
New referrals	4 178	3 740	4 315	3 850	-7.9
Control consultatiens	7 030	7 929	9 162	10 689	+52.0
***Rheumatology***					
Total admittance	522	453	571	606	+16.1
Emergency	94	59	103	109	+16.0
Elective	428	394	468	497	+16.1
Outpatient visits, total	2 601	2 466	3 854	3 838	+47.6
New referrals	733	813	1.105	811	+10.6
Control consultatiens	1 868	1 653	2 718	3 027	+62.0
***Dermatology***					
Total admittance	266	335	342	508	+91.0
Emergency	88	74	96	152	+72.7
Elective	175	261	246	356	+203.6
Outpatient visits, total	25 046	19 927	19 260	19 824	-20.9
New referrals	3 781	2 876	2 847	2 766	-26.8
Control consultatiens	21 265	17 051	16 413	17 058	-19.8
***Neurology***					
Total admittance	1 041	1 103	1 233	1 421	+36.5
Emergency	771	893	999	938	+21.7
Elective	270	210	234	483	+78.9
Outpatient visits, total	5 826	6 813	5 949	6 278	+7.8
New referrals	3 735	4 302	3 728	4 083	+9.3
Control consultatiens	2 091	2 511	2 221	2 195	+5.0

**Table 5:** Admitted patients, operations and outpatient consultations and case-mix at selected Surgical departments at St. Olavs University Hospital during the time period 1995 to 2001.

Department	1995	1997	1999	2001	Change (%)
***General surgery***					
Total admittance	5 542	5 807	5 954	6 198	+11.8
Emergency	2 882	3 012	3 096	3 347	+16.1
Elective	2 660	2 795	2 858	2 851	+7.2
Operations	6 053	6 800	6 328	6 547	+8.2
Out-patients visits, total	16 193	18 988	17 178	20 672	+27.7
New referrals	7 600	7 816	5 884	5 914	-21.2
Control consultations	8 593	11 172	11 294	14 758	+71.7
***Orthopedic surgery***					
Total admittance	4 691	4 748	4 646	5 068	+8.0
Emergency	2 392	2 659	3 020	2 838	+18.6
Elective	2 299	2 089	1 626	2 230	-3.0
Operations	5 662	5 766	5 404	5 825	+3.4
Out-patients visits, total	20 120	20 912	22 732	22 866	+13.6
New referrals	6 710	7 438	7 018	5 719	-14.8
Control consultations	13 410	13 474	15 705	17 147	+27.9
***Neurosurgery***					
Total admittance	1 620	1 634	1 567	1 826	+12.7
Emergency	923	1 046	956	745	-19.3
Elective	697	588	611	1 081	+55.1
Operations	976	935	848	1 199	+22.8
Out-patients visits, total	2 104	2 301	2 696	3 260	+54.9
New referrals	646	807	1 050	1 304	+101.8
Control consultations	1 458	1 494	1 646	1 956	+34.1
***Gynecology***					
Total admittance	1 997	1 899	1 825	1 807	-9.5
Emergency	799	760	748	560	-29.9
Elective	1 198	1 139	1 077	1 247	+4.1
Operations	2 680	2 595	2 365	2 395	-10.6
Out-patients visits, total	14 359	13 085	14 071	13 717	-4.5
New referrals	10 686	10 036	9 627	7 792	-27.1
Control consultations	3 673	3 049	4 444	5 925	+61.3
***Otorhinolaryngeology***					
Total admittance	1 223	1 332	1 497	1 282	+0.07
Emergency	318	373	389	410	+28.9
Elective	905	959	1 108	872	-3.6
Operations	1 766	1 895	1 812	1 745	-1.2
Out-patients visits	11 207	11 186	11 535	12 472	+11.3
New referrals	4 097	5 593	5 263	4 795	+17.0
Control consultations	7 110	5 593	6 272	7 677	+8.0
***Ophthalmology***					
Total admittance	1 185	1 069	1 065	853	-28.0
Emergency	675	567	607	426	-37.0
Elective	510	502	458	427	-16.3
Operations	1 749	2 208	2 416	2 395	+36.9
Out-patients visits	15 313	13 953	16 477	17 135	+11.9
New referrals	4 888	5 254	5 267	3 312	-33.2
Control consultations	10 425	8 699	11 210	13 823	+32.6

**Table 6:** Outcome and quality related data at St. Olavs University Hospital during the time period 1995 to 2001.

	**1995**	**1997**	**1999**	**2001**	**Change %**
Patients in high risk DRG groups^$^	2 629	2 435	2 555	2 548	-3.1
In hospital deaths in these	184	200	238	205	+11.4
Laboratory tests/patient^β^	33.4	34.2	32.4	29.2	-12.6
Imaging examinations/patient^δ^	3.7	3.2	3.1	3.2	-15.6

^$^includes Norwegian DRG's 14, 89–90. 121–122, 127, 210–211, 236

^β^includes all laboratory tests/procedures excluding pathology (data not available) on in-hospital patients.

^δ^includes imaging examinations on in-hospital patients.
